# The Transcriptional Response of *Aedes aegypti* with Variable Extrinsic Incubation Periods for Dengue Virus

**DOI:** 10.1093/gbe/evy230

**Published:** 2018-10-18

**Authors:** Cassandra Koh, Scott L Allen, Rosemarie I Herbert, Elizabeth A McGraw, Stephen F Chenoweth

**Affiliations:** 1 School of Biological Sciences, Monash University, Melbourne, Victoria, Australia; 2 Department of Entomology, Center for Infectious Disease Dynamics, Pennsylvania State University, University Park, PA, United States; 3 School of Biological Sciences, The University of Queensland, Brisbane, Queensland, Australia

**Keywords:** dengue virus, mosquito, Aedes, extrinsic incubation period, transcriptome

## Abstract

Dengue fever is the most prevalent arboviral disease globally. Dengue virus is transmitted primarily by the *Aedes aegypti* mosquito. One measure of the mosquito’s efficiency as a vector is the extrinsic incubation period (EIP), which is the time between the ingestion of viremic blood and the emergence of virions in the saliva. The longer it takes virus to infect the midgut and traverse to the saliva, the fewer opportunities the mosquito will have to transmit the pathogen over its lifetime. We have shown previously that EIP for dengue virus is highly heritable and that it is negatively correlated with vector lifespan. Here, we examined the transcriptional profiles for mosquitoes that varied in their EIP phenotype and identified pathways associated with either short or long EIP. We found that mosquitoes with short EIP have less active immune responses but higher levels of protein translation and calcium ion homeostasis and that mosquitoes with longer EIP may have slower metabolism. These findings indicate a complex interplay between calcium ion distribution, ribosome biogenesis, and metabolism and reveal potential pathways that could be modified to slow the rate of viral progression and hence limit lifetime transmission capability.

## Introduction

Dengue virus is the causative agent of dengue fever and the life-threatening dengue hemorrhagic fever or dengue shock syndrome. Between 100 and 390 million new infections occur every year (Bhatt et al. 2013) with an estimated 3.97 billion people at risk of infection in 128 countries across tropical and subtropical regions, making dengue virus one of the most clinically important arboviruses worldwide (Brady et al. 2012). This positive-sense single-stranded virus comprises four antigenically distinct serotypes and is a member of the genus *Flavivirus*, which also includes other clinically important human pathogens such as West Nile virus, yellow fever virus, and Japanese encephalitis virus (Mackenzie et al. 2004). Dengue virus is primarily transmitted to humans by the mosquito vector *Aedes aegypti*. These mosquitoes are especially efficient vectors as they are well-adapted to living in close vicinity to humans, preferring to lay eggs in stagnant water bodies around densely populated urban areas (Mackenzie et al. 2004). Increasing urbanization is therefore assisting with the global expansion and spread of dengue fever (Bhatt et al. 2013).

Mosquitoes are not passive participants in the transmission cycle, as the virus must actively infect a range of mosquito tissues to be successfully transmitted. Following consumption of an infectious blood meal from a human, the virus must first infect the midgut. This tissue serves as a strong barrier, and some infections will not progress beyond this tissue. Viruses escaping the midgut must traverse tissues in the body cavity until they reach the salivary glands. Once this tissue is infected, virions can be secreted into the saliva and be transmitted to a human on a subsequent bite (Black et al. 2002). The time between the ingestion of a viremic blood meal and the emergence of virions in the saliva is termed the extrinsic incubation period (EIP) (Schule 1928). Mathematically, EIP in combination with longevity represents the most powerful determinant of the number of infectious bites a mosquito can induce (Macdonald 1957). This is because the earlier the virus arrives in the saliva the more opportunities the mosquito will have to infect a human over its lifetime. This is particularly the case for *A. aegypti* who are known to seek blood meals every few days (Mackenzie et al. 2004). It also means that if EIP could be delayed through targeted genetic modification, the impact would be far greater than reducing susceptibility.

The measure of how efficiently an insect vector can transmit a pathogen is known as vector competence (VC) (Hardy et al. 1983). It is determined by a combination of environmental and genetic factors, such as temperature, mosquito nutrition, and viral and mosquito genotypes (Gubler et al. 1979; Watts et al. 1987; Black et al. 2002; Bara et al. 2015). Mosquito populations exhibit higher VC for viral strains that are sampled from the same geographic region, likely reflecting a history of coadaptation between mosquito and virus (Lambrechts et al. 2009). Most of the studies examining the genetic basis of VC have focused on a few key traits as a means to measure susceptibility. These traits are commonly defined by virus infectivity of the midgut or tissues beyond, such as fat bodies or legs, as an indicator of dissemination from the midgut (Salazar et al. 2007; Lambrechts et al. 2011). Detection of virus in the head or salivary glands has been used as a proxy measure for EIP (Bennett et al. 2002; Carrington et al. 2013). However, the presence of virus in secreted saliva is the most accurate measure of vector transmissibility (Ye et al. 2015).

Recently, work from our group examined the genetic architecture of EIP as measured by repeat sampling of the saliva of individual mosquitoes within a family breeding design (Ye, Chenoweth, et al. 2016). We showed that EIP was highly heritable (*H*^2^ = 0.38) and hence should have the capacity to adapt in wild populations. We also revealed a positive genetic correlation between EIP length and mosquito lifespan (Ye, Chenoweth, et al. 2016). The nature of this relationship could be explained in one of two ways. First, mosquitoes with shorter EIP lengths may simply be less fit and hence unable to slow the rate of viral replication in their bodies. Alternatively, pleiotropy may generate tradeoffs between survival and viral control, with good controllers of virus suffering the cost of such activities.

Horizontal or vertical infection by dengue virus is mildly virulent to the mosquito vector, causing decreased longevity, slower growth, and reduced fecundity (Maciel-de-Freitas et al. 2011; Sylvestre et al. 2013). Thus, evolutionary pressure is predicted to select for mosquito genotypes that confer better resistance or tolerance against pathogen infection (Hurd et al. 2005). The rarity of DENV in mosquito populations (Adams and Boots 2010), however, suggests that this selection pressure might be small, whereas the near constant presence of insect-specific flaviviruses in mosquito populations (reviewed by Bolling et al. [2015]) could offer a means to maintain such selection. Regardless of mechanism, genetic variation for dengue resistance appears to be maintained, suggesting a trade-off between resistance to dengue and fitness, possibly due to increased energetic costs from mounting an immune response (Sheldon and Verhulst 1996). Although there are currently no empirical studies demonstrating fitness costs accompanying higher resistance to dengue in *Aedes* vectors, studies in *Drosophila* and *Anopheles* systems have showed that fitness or reproductive cost can occur with the evolution of resistance against pathogens (Hurd et al. 2005; Ye et al. 2009).

Dengue infection in *A. aegypti* triggers changes in gene expression patterns that can be both host- and virus-driven. Host-driven transcriptomic changes are related to innate immune defenses as seen in the upregulation of genes involved in multiple immune pathways: TOLL, IMD, and JAK-STAT immune signaling pathways, as well as the RNA interference pathway (Xi et al. 2008; Sanchez-Vargas et al. 2009; Sim et al. 2013). These pathways are effective in limiting virus replication. The intensity of these transcriptional responses to infection determines the degree of resistance against the virus (Ocampo et al. 2013). Higher basal expression of immunity-related genes also confers increased resistance against the virus (Sim et al. 2013). There is evidence of virus-driven downregulation of immune-related genes, possibly as an adaptation to evade immune responses and assist viral survival (Sim and Dimopoulos 2010; Colpitts et al. 2011; Bonizzoni et al. 2012). Upregulation of host genes involved in metabolic processes has also been observed, which may support viral replication by increasing energy production or accumulating resources required for replication, such as lipids (Heaton et al. 2010). Thus, the mosquito transcriptome during dengue infection at any point in time is the product of two sources of modulation: the host and the virus. To date, no studies have compared transcriptional landscapes of mosquitoes with different EIP lengths, which may offer insights into the intracellular environments that lead to extreme EIP phenotypes.

To understand whether there are biological processes in the mosquitoes that may underpin differences in EIP, we conducted transcriptome sequencing on mosquitoes exhibiting EIP lengths between 6 and 12 days postinfection (DPI) and found highly diverse differentially regulated genes. Through functional analyses, we discuss biological processes that may underpin the EIP phenotype.

## Materials and Methods

### Mosquito Collections and Rearing

*Aedes* *aegypti* eggs were collected from Cairns, QLD, Australia using ovitraps as in Ye, Chenoweth, et al. (2016) and reared in standard laboratory conditions established in Moreira et al. (2009). Briefly, hatched *A. aegypti* larvae were reared in 30 cm × 40 cm × 8 cm plastic trays at a standard density of 150 individuals per 3 l of distilled water and fed with fish food pellets (Tetramin Tropical Tablets, Tetra, Melle, Germany) until pupation. Pupae were collected and transferred to 30 cm × 30 cm × 30 cm cages for eclosion at a density of 450 individuals per cage. Posteclosion, adults were fed with 10% sucrose solution ad libitum. Mosquitoes at all stages were maintained in a controlled environment insectary at 25 °C, 65% humidity, and on a 12h:12 h light:dark cycle.

### Virus Culture

Dengue virus serotype 3 (DENV-3), isolated from a patient during an outbreak in Cairns in 2008/2009, was used in this study (Ritchie et al. 2013). Virus was propagated in *Aedes albopictus* C6/36 cells for eight passages to generate high titer for infection as in Frentiu et al. (2010). Briefly, cells were inoculated with virus when they formed a confluency of 80% in a monolayer and maintained on RPMI 1640 media supplemented with 2% heat-inactivated fetal bovine serum (Life Technologies, Carlsbad, CA), 1% Glutamax (Life Technologies), and 25 mM HEPES buffer (Sigma Aldrich) at 26 °C. At 7 DPI, virus was harvested by centrifugation of the cell culture media at 3,200 × g at 4 °C to obtain a viremic supernatant to be used immediately for oral infection of mosquitoes.

### Transcriptomic Collections

We sought to determine whether mosquitoes that varied for EIP had particular transcriptomic patterns. Mosquitoes were reared in populations and experiments carried out three generations after the original field collection. A total of four hundred 6- to 8-day-old females were fed viremic blood (Treatment = DENGUE) and assessed for EIP as per below. To control for effects of blood-feeding and age, 250 age-matched female mosquitoes were fed with nonviremic blood on the same day (Treatment = BLOOD). When a mosquito’s saliva tested positive for virus, the subsequent day the whole body of that individual was collected, homogenized, and total RNA was extracted using TRIzol reagent as per the manufacturer’s instructions (Life Technologies). RNA samples reconstituted in RNase-free water were then treated with DNase I recombinant enzyme (Roche, Basel, Switzerland) according to the manufacturer’s protocol to eliminate genomic DNA contamination. On the same day, ten whole bodies each from the BLOOD treatment were collected and extracted as above as controls to match the collection timepoint.

### EIP Measurement

The EIP assay was carried out as previously reported (Ye et al. 2015). In brief, mosquitoes were prepared for oral infection by starving for at least 24 h. Mosquitoes were offered a 1:1 (v/v) mixture of defibrinated sheep blood and live DENV-3 supernatant at a concentration of 1 × 10^5^ plaque-forming units/ml as measured by plaque assay (Ye et al. 2015). This blood–virus mixture was maintained at 37 °C during the feed with the use of a water-jacketed feeding apparatus. Mosquitoes were allowed to feed through a membrane of porcine intestine. Blood-engorged mosquitoes were sorted under CO_2_ and housed individually in 70-ml polypropylene containers sealed with a piece of polyester mesh and provided with 200 μl of 10% (w/v) sucrose to feed on. If virus dissemination has reached the salivary glands from the midgut, viral particles will be secreted with saliva when the mosquito feeds from the sucrose solution (Hall-Mendelin et al. 2010). The sucrose solutions from each container were collected every 2 days from 5 DPI for the breeding experiment and from 6 DPI for the transcriptomic collections. For the breeding experiment, viral RNA was purified from sucrose solution samples using the PureLink Pro 96 Viral RNA/DNA Kit (Invitrogen, Carlsbad, CA). Viral RNA was then reverse-transcribed with SuperScript III (Invitrogen) to generate cDNA for quantitative real time polymerase chain reaction (qRT-PCR) using methods described by Moreira et al. (2009) and Richardson et al. (2006). For transcriptomic collections, sucrose solution samples were mixed with 50 μl of extraction buffer (10 mM Tris pH 8.2, 1 mM ethylenediaminetetraacetic acid, 50 mM NaCl, and 1.25% v/v proteinase K [Yeap et al. 2014]) and incubated in a thermocycler block at 56 °C for 5 min followed by 98 °C for 5 min (Yeap et al. 2014). DENV genome copies were quantified in samples by qRT-PCR with TaqMan Fast Virus 1-Step Master Mix (Thermo Fisher Scientific, Waltham, MA) according to the manufacturer’s protocol (Ye, Carrasco, et al. 2016). The primers, probes, and DENV standards used here have been previously described (Warrilow et al. 2002). The first day of detection of dengue viral RNA in saliva samples indicated the successful dissemination of virus from midgut into saliva and was scored as EIP.

### Transcriptomic Sequencing

A total of six individual mosquitoes with each of the following EIP measurements; 6, 8, 10, and 12 DPI from each treatment (BLOOD and DENGUE) were extracted for total RNA. All the samples passed standard RNA quality control, thus forty-eight 100-bp paired-end libraries were prepared with the Illumina TruSeq Stranded mRNA-seq LT library prep kit, which included an mRNA purification step using poly-T beads and sequenced for 50 cycles with the Illumina HiSeq2000 platform. A complete block design was employed for sequencing (Auer and Doerge 2010), where sequencing lane was treated as an experimental block. This design was technically replicated across flowcells so that each library was essentially sequenced twice, once in a different lane of each flowcell. Quality control of the sequence data revealed that a single 6 DPI DENGUE sample (D_6DPI_3_CGGCTATG) had a relatively high proportion of adapter sequence. For this reason, adapter sequence was trimmed from all the samples using the ILLUMINACLIP option in Trimmomatic (version 0.32) (Bolger et al. 2014) with the following standard settings, two mismatches in the adapter alignment were allowed, a simple alignment score threshold of 10, and a palindrome alignment score threshold of 30. Trimmed reads that were <50 bp were discarded which resulted in the loss of ∼10% of reads from sample D_6DPI_3_CGGCTATG, whereas all other samples lost ∼1% of reads.

Reads were mapped to the *A. aegypti* Liverpool LVP strain genome AaegL3 obtained from VectorBase (Giraldo-Calderon et al. 2015) using HiSat2 (version 2.04) (Kim et al. 2015). This genome consists of 4,757 scaffolds spanning 1.3 GB; it has been annotated with 15,796 genes and 18,840 transcripts. An average 75% of reads were successfully mapped, and the number of reads per gene was counted using featureCounts from subreads version 1.5.1 (default settings) (Anders et al. 2014). A gene was deemed as expressed if at least 24 samples had greater than one count-per-million (CPM) as calculated using HTSFilter in R (Rau et al. 2013), 11,897 genes passed this expression threshold, and the remaining 3,899 genes were excluded from further analysis. To account for unknown systemic variation generated during RNA extraction, library preparation, and sequencing, surrogate variable analysis was performed using the SVAseq package in R (Leek and Storey 2007; Leek 2014), which produced ten cofactors used in the model below. Expression counts were converted to log-CPM and normalized using TMM as is default for the estimation of VOOM precision weights, which were used to permit analysis of RNAseq data using a general linear model in LIMMA (Smyth 2004; Law et al. 2014).

To test for a significant relationship between EIP and DENV infection that was not a response to blood-feeding, the following linear model was used:

(1) expression = sample + SV1 + SV2 + SV3 + SV4 + SV5 + SV6 + SV7 + SV8 + SV9 + SV10 + EIP + treatment + EIP × treatment,

where sample was a random effect, SV1-10 represents ten surrogate variables fit independently as fixed, EIP was the categorical fixed effect of DPI that DENV was detected in saliva, and treatment was a fixed effect with two levels (DENGUE or BLOOD). A significant interaction between EIP and treatment indicated that the differences in expression for each EIP group were not the same for DENGUE and BLOOD mosquitoes. All models were run using LIMMA in R (Ritchie et al. 2015).

### Gene Ontology Term Enrichment Analysis

To examine what biological processes are affected by dengue infection, we conducted Gene Ontology (GO) term enrichment analysis using g:Profiler (Reimand et al. 2007). Functions of member genes in significant GO terms were identified by searching gene IDs against VectorBase. Functions of genes with no known function in VectorBase were inferred by searching for their *Drosophila melanogaster* orthologs against FlyBase.

### RNAi Knockdown

A group of genes associated with the term GO:0006412 *translation* was very significantly enriched among genes differentially regulated by an interaction of EIP and dengue infection. Given viral dependence on host cell translation machinery, we selected genes from this GO term for manipulation through RNAi knockdown. To create dsRNAs, templates 300–600 bp long were synthesized with Q5 High-Fidelity DNA Polymerase (New England BioLabs, Ipswich, MA) from *A. aegypti* cDNA using primer pairs designed to target the coding sequences of each candidate gene (Kulkarni et al. 2006). Primers were designed using the Primer-BLAST online tool (Ye et al. 2012) and were linked with a T7 promoter sequence at the 5′ end (TAATACGACTCACTATAGGGAGACCAC) (Kennerdell and Carthew 1998). PCR product size and purity were verified through gel electrophoresis and isolated with MinElute PCR Purification Kit (Qiagen, Hilden, Germany). Templates were then used to produce dsRNA using the MEGAscript T7 Transcription Kit (Ambion, Foster City, CA) and lithium-chloride precipitated as per the manufacturer’s instructions. The immune-competent *A. aegypti* Aag-2 cell line (Barletta et al. 2012) was used to validate the effect of RNAi knockdown on virus replication using methods as described by Terradas et al. (2017). Briefly, Aag-2 cells were seeded in a 96-well plate at 80% confluence 24 h prior to transfection. Transfections were performed with Lipofectamine RNAiMAX transfection reagent (Invitrogen) according to manufacturer’s instructions with 1 pmol of dsRNA per well in serum-free media.

To measure gene expression after dsRNA treatment, RNA was isolated from Aag-2 cell pellets using TRIzol reagent (Life Technologies) as described above. cDNA was synthesized using SuperScript III Reverse Transcriptase (Invitrogen) with random primers (Thermo Fisher Scientific) following the manufacturer’s protocol. Gene expression levels were quantified by qRT-PCR with SYBR Green I (Roche). Thermalcycling conditions are as per manufacturer’s protocol. qRT-PCR primers of each candidate genes were again designed using Primer-BLAST (Ye et al. 2012). All candidate genes were run in parallel with a housekeeping gene *rpS17*. Normalized expression was calculated using the method described by Simon (2003). The effect of RNAi knockdown on gene expression levels was analyzed using Mann–Whitney *U* tests with GraphPad Prism 7 software (San Diego, CA) with level of significance set at *P* < 0.05.

To investigate the effect of candidate gene knockdown on DENV replication, cells transfected with dsRNA were inoculated 24 h later with DENV-3 at a multiplicity of infection of 0.01 for 2 h (Terradas et al. 2017). Infected cells were maintained on complete media supplemented with 2% fetal bovine serum. At 6 DPI, media containing released virions were collected from wells for quantification of virus titer by qRT-PCR (as per above). The collection point was based on pilot studies and previous work from our group (Terradas et al. 2017) showing that the dsRNA knockdown lasted for approximately 7 days and hence we were seeking to capture the cumulative impact of that period on DENV replication. DENV-3 titers between dsRNA-treated cells and control cells were analyzed using a Kruskal–Wallis test followed by Dunn’s multiple comparison test with GraphPad Prism 7 software with level of significance set at *P* < 0.05.

## Results

### Transcriptional Profiling of Mosquitoes with Variable EIP

We first sought to understand the physiological mechanisms driving variation in EIP by identifying associated patterns in transcriptomic responses. Following blood feeding of virus (treatment = DENGUE) to populations of mosquitoes, we characterized individual mosquitoes for their day of virus arrival in the saliva across 4 days: 6, 8, 10, or 12 DPI. Mosquitoes were collected for transcriptional profiling the day after their saliva became positive for virus. The main effect of treatment in model (1) was significant for 258 genes at a false discovery rate (FDR) of 5%, which represent general transcriptomic changes in response to dengue irrespective of DPI. Among these genes, a total of 55 GO terms were found to be significantly enriched using g:Profiler (supplementary table 1, Supplementary Material online). Forty-eight of the enriched GO terms (FDR 5%) were related to metabolic or biosynthetic processes. Additionally, we found one GO term associated with oxidation–reduction and six GO terms associated with ATP generation.

To understand what dengue-triggered changes in biological processes might specifically underpin the EIP phenotype, we investigated GO biological process terms that were enriched among genes associated with a significant interaction between dengue infection and EIP length in model (1). A total of 182 genes were significant for the interaction at an FDR of 5% and among these there were 73 significantly enriched GO terms (FDR 5%) (supplementary table 2, Supplementary Material online). Several GO terms were particularly noteworthy given their patterns of expression change across the four EIP groups; GO:0006952 *defense response* (*P* = 0.0041), GO:0022900 *electron transport chain* (*P* = 0.00144), GO:0070588 *calcium ion transmembrane transport* (*P* = 0.0364), and GO:0006412 *translation* (*P* = 2.4 × 10^−33^).

**Fig. 1.**
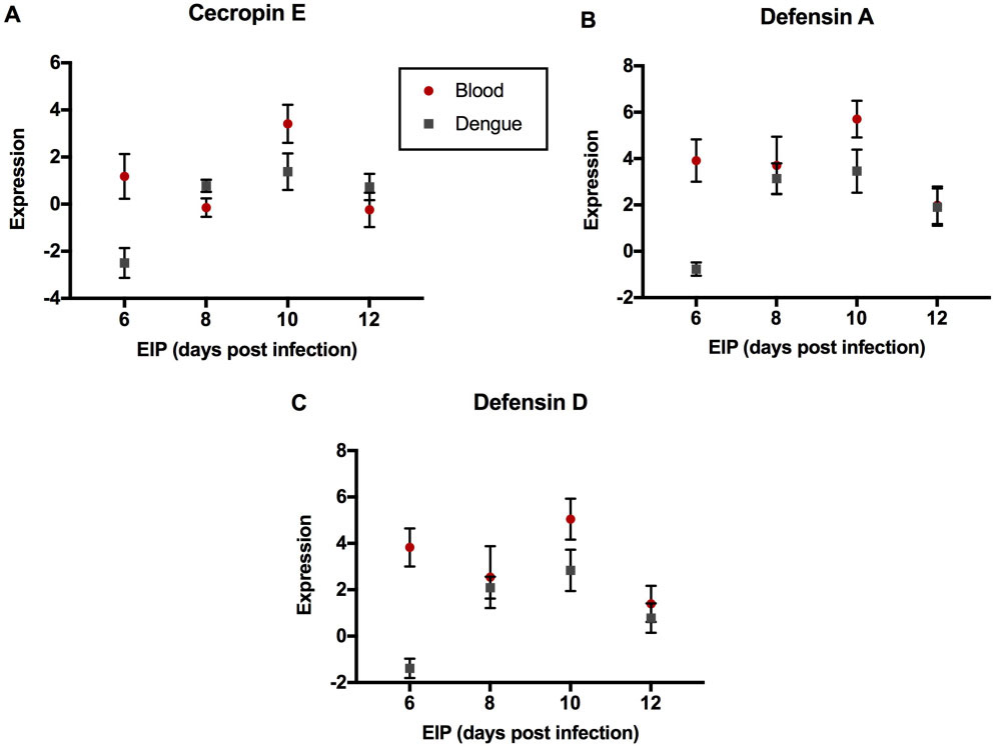
—Genes of enriched GO term GO:0006952 *defense response*. Expression levels by EIP phenotype of (*A*) *Cecropin E*, (*B*) *Defensin A*, and (*C*) *Defensin D* are shown. Expression levels were estimated from read counts from RNAseq data. Red circles depict mosquitoes fed with a naïve blood meal, while gray squares depict those fed with a viremic blood meal. Mean and SEM are shown in graphs (*n* = 6 per group per treatment).

**Fig. 2.**
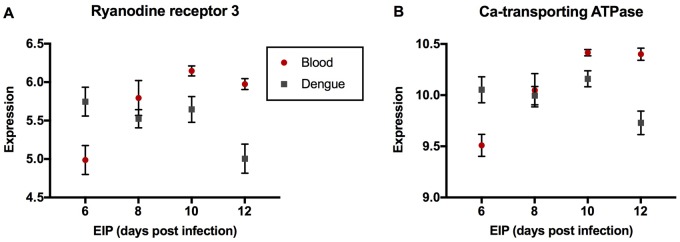
—Genes of enriched GO term GO:0070588 *calcium ion transmembrane transport*. Expression levels by EIP phenotype of (*A*) *Ryanodine receptor 3* and (*B*) *calcium-transporting ATPase* are shown. Expression levels were estimated from read counts from RNAseq data. Red circles depict mosquitoes fed with a naïve blood meal, while gray squares depict those fed with a viremic blood meal. Mean and SEM are shown in graphs (*n* = 6 per group per treatment).

**Fig. 3.**
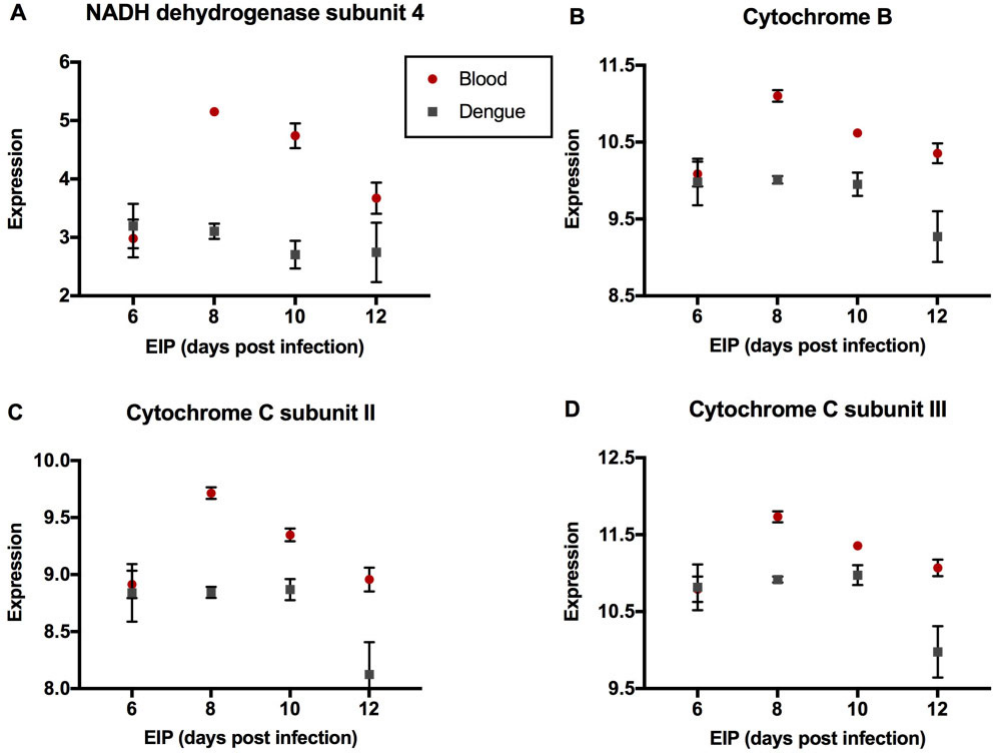
—Genes of enriched GO term GO:0022900 *electron transport chain*. Expression levels by EIP phenotype of (*A*) *NADH dehydrogenase subunit 4*, (*B*) *cytochrome B*, (*C*) *cytochrome C subunit II*, and (D) *cytochrome C subunit III* are shown. Expression levels were estimated from read counts from RNAseq data. Red circles depict mosquitoes fed with a naïve blood meal, while gray squares depict those fed with a viremic blood meal. Mean and SEM are shown in graphs (*n* = 6 per group per treatment).

**Fig. 4.**
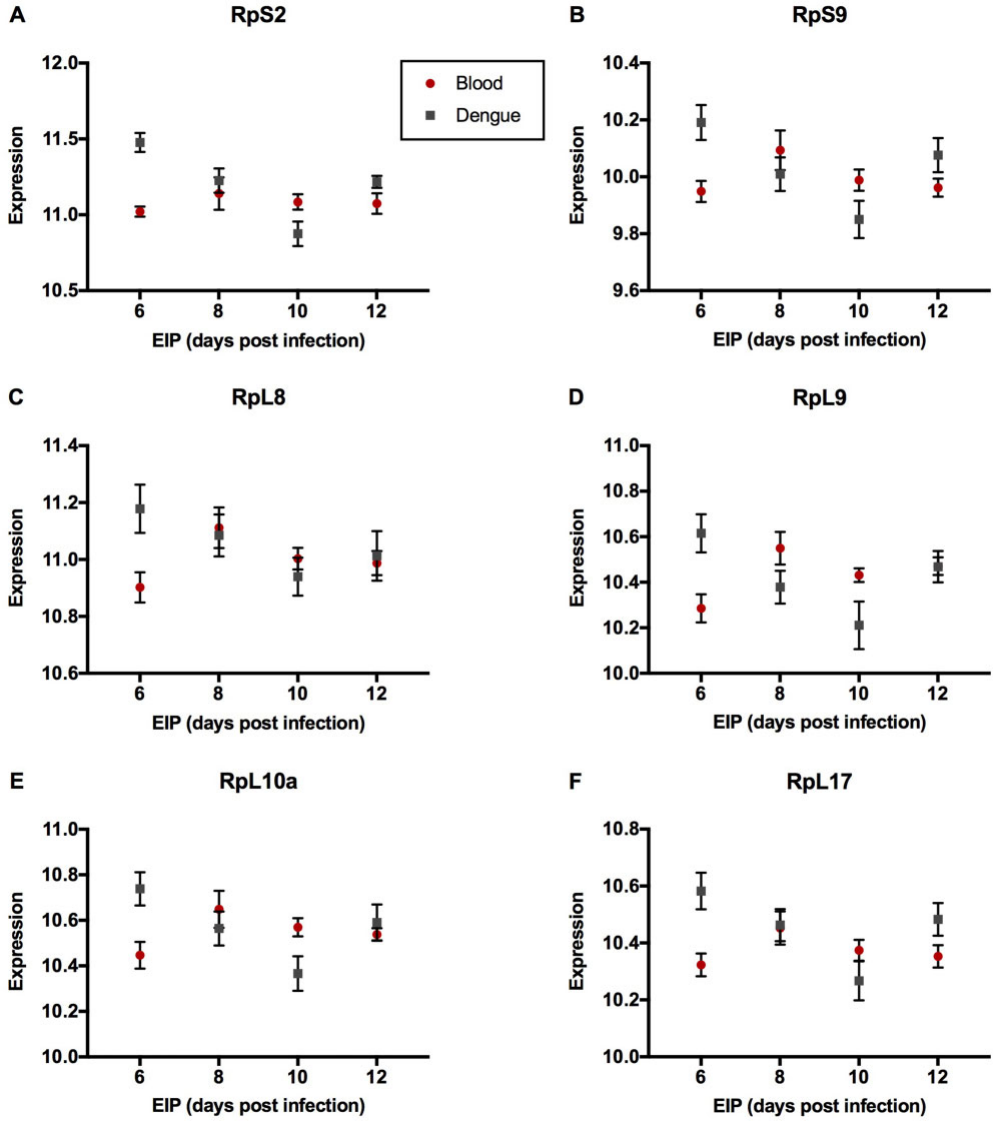
—Genes of enriched GO term GO:0006412 *translation*. Expression levels by EIP phenotype of (*A*) *RpS2*, (*B*) *RpS9*, (*C*) *RpL8*, (*D*) *RpL8*, (*E*) *RpL10a*, and (*F*) *RpL17* are shown. Expression levels were estimated from read counts from RNAseq data. Red circles depict mosquitoes fed with a naïve blood meal, while gray squares depict those fed with a viremic blood meal. Mean and SEM are shown in graphs (*n* = 6 per group per treatment).

The GO:0006952 *defense response* group comprises three genes—AAEL000611 (*Cecropin E*), AAEL003841 (*Defensin A*), and AAEL003857 (*Defensin D*), that were expressed at a lower level in DENGUE relative to BLOOD mosquitoes in 6 and 10 DPI groups but not in 8 and 12 DPI groups (fig. 1). As the downregulation in DENGUE mosquitoes was stronger in the 6 DPI group, this may indicate an importance of a diminished immune response for rapid virus dissemination. The GO:0070588 *calcium ion transmembrane transport* group comprises two calcium transporters bound to the endoplasmic reticulum (ER) membrane. AAEL003837 (*ryanodine receptor 3*) encodes a channel protein that releases stored calcium ions from the ER lumen into the cytoplasm (Meissner 1994), and AAEL006582 (*calcium-transporting ATPase*) is an ATP-dependent pump which maintains low cytosolic concentrations of calcium ions by actively transporting them into the ER (Clapham 1995). Despite their antagonistic functions, both had higher expression in DENGUE relative to BLOOD mosquitoes in the 6 DPI group, but lower expression in 10 and 12 DPI groups (fig. 2). The GO:0022900 *electron transport chain* group includes AAEL018658 (*NADH dehydrogenase subunit 2*), AAEL018664 (*cytochrome C subunit II*), AAEL018669 (*cytochrome C subunit III*), and AAEL018685 (*cytochrome B*). These genes were uniformly expressed lower in DENGUE relative to BLOOD mosquitoes in 8, 10, and 12 DPI groups (fig. 3). It is not yet clear whether this decrease in energy production would benefit viral replication or host defenses. GO:0006412 *translation* is a larger group containing 41 genes, most of which codes for ribosomal components. Here, we show several representative genes—AAEL010168 (*RpS2*), AAEL001759 (*RpS9*), AAEL000987 (*RpL8*), AAEL007699 (*RpL9*), AAEL013221 (*RpL10a*), and AAEL011471 (*RpL17*)—all of which were strongly upregulated in DENGUE relative to BLOOD mosquitoes in 6 DPI but not in later EIP groups (fig. 4). It can be expected that higher protein synthesis efficiency would promote faster viral replication.

### dsRNA Silencing of Ribosomal Components Does Not Affect Dengue Titer

Among the GO terms identified in our analyses of genes differentially affected by dengue, the GO:0006412 *translation* group was one of the most significantly enriched terms and consisted of mostly ribosomal subunit proteins. Given that viral replication is highly dependent on host translational machinery, we sought to shift the EIP phenotype through manipulation of the expression of genes under this GO term. To achieve this, we selected four candidate genes and used RNA interference to deplete their expression levels and observe for changes in viral titer in Aag-2 cells. dsRNAs were designed to target the coding sequences of four ribosomal genes—*RPS2*, *RPS9*, *RPL9*, and *RPL10a*. Strong and persistent knockdown was achieved for only three genes: RPS9, RPL9, and RPL10 (supplementary fig. 1, Supplementary Material online). The knockdown effect of these four genes on DENV replication was investigated by inoculating Aag-2 cells with DENV-3 at 1-day posttransfection with dsRNA. Six days after inoculation with DENV, the titer of DENV in the cell culture supernatant was determined through qRT-PCR. We compared DENV copy numbers from dsRNA-treated cells to a mock-transfected control with Student’s *t*-tests and found no significant effect of any of the three dsRNAs on DENV titer in the supernatant (*P *> 0.05) (supplementary fig. 2, Supplementary Material online).

## Discussion

When we compared the transcriptomes of DENGUE and BLOOD mosquitoes irrespective of EIPs, the enriched GO terms include those associated with metabolic processes, redox functions and ATP generation. These findings are in keeping with other transcriptomic studies on the effects of DENV infection in *A. aegypti* (Xi et al. 2008; Colpitts et al. 2011; Bonizzoni et al. 2012; Raquin et al. 2017). Our specific goal in this study, however, was to identify a set of genes that underpin DENV EIP in mosquitoes. By sampling individuals with particular EIPs, rather than all mosquitoes at a given DPI, our candidate list should be biased toward EIP-associated genes. Our list of candidates, however, may also contain some genes with signatures of time-dependent expression during DENV infection that do not directly affect EIP. These two possibilities will need to be differentiated with further functional studies for the genes of interest.

It is not surprising that our screen identified antimicrobial peptide genes. The production of AMPs has previously been shown to change during the time course of infection (Bonizzoni et al. 2012). All the candidates in this case, *Cecropin E*, *Defensin A*, and *Defensin D*, are encoded by the TOLL pathway, activated by the presence of ROS during infection with DENV (Sabin et al. 2010). The importance of TOLL in limiting DENV infection has been previously demonstrated in both *D.melanogaster* and *A. aegypti* (Zambon et al. 2005; Xi et al. 2008). The specific mechanism of how these antimicrobials operate against DENV in insects is unknown, although human defensins have been shown to inhibit a range of key steps in viral replication and assembly (Ding et al. 2009). It is possible that mosquitoes with shorter EIP may possess a weaker inducible immune response rendering them less able to slow virus proliferation. Our own work has previously demonstrated the cost to insects for activating innate immunity pathways like TOLL (Ye et al. 2009). There is also substantial evidence that insects trade off growth (Wang et al. 2005; Becker et al. 2010) and reproduction (McKean and Nunney 2005) against immunity (reviewed by Howick and Lazzaro [2014]).

Calcium ions play a role in intracellular signaling in the host (Berridge et al. 2000). Previous studies have demonstrated that during DENV infection the concentration of calcium ions rises in the cytoplasm (Chen et al. 2017) as levels are simultaneously depleted in the ER (Dionicio et al. 2018). These changes in calcium ion distribution are associated with oxidative stress (Mbaya et al. 2010; Hammadi et al. 2013) and may simply be a consequence of ER remodeling (Mackenzie et al. 2004). Alternatively, they may represent a specific manipulation of the host given that when calcium ion channels are blocked or if calcium is chelated, fewer infectious viruses are created (Dionicio et al. 2018). Also, oxidative stress and calcium misdistribution trigger autophagy (Datan et al. 2016), and autophagy and prolonged cell survival are in turn favorable for viral replication (McLean et al. 2011). In 6 DPI mosquitoes, we see the increased expression relative to BLOOD of two different calcium ion transport-associated genes that act in opposition to one another. *Ryanodine receptor 3* releases ions from the ER lumen into the intracellular space (Meissner 1994), and a *calcium-transporting ATPase* assists the movement of calcium ions from the cytoplasm into the ER (Clapham 1995). The former may be caused by infection, while the latter is likely to be a host counter response. Interestingly, in 10 and 12 DPI mosquitoes, the expression of these genes is lower than in BLOOD which may be expected as the infections have progressed more slowly and potentially with lower viral replication in these insects.

Interestingly, several previous studies have revealed that DENV causes the shutdown of host cell translation activities and in so doing protects cell survival (Hou et al. 2017; Dionicio et al. 2018). In response to calcium misdistribution and oxidative stress as per above, autophagy is activated by the removal of suppression on the mammalian *target of rapamycin* (*mTor*) pathway (Datan et al. 2016). A side effect of this removal is the shutdown of cap-dependent translation, an effect that may benefit viral replication by making host cell translation machinery more available (Foster and Fingar 2010). Mosquitoes with short EIPs exhibited higher rates of ribosomal gene transcription and that may be speeding the proliferation of virus by increasing availability of ribosomes, but also leading to greater cell harm. Like for the AMP-encoding genes, the expression of ribosomal genes has been shown to change during infection (Raquin et al. 2017).

In mosquitoes with longer EIP >6 DPI, we saw decreased expression of four genes associated with the electron transport chain, including genes that produce subunits of cytochromes B and C and NADH dehydrogenase. The electron transport chain and its activities produce ATP for the cell and hence set the pace for cellular activities including ribosome biogenesis and protein synthesis more broadly (MacInnes 2016). It is possible that cells producing less ATP are able to slow the rate of viral replication and proliferation. Aside from being the main generators of ATP, the mitochondria also produce ROS (Ferguson et al. 2005). As per above, ROS triggers defense responses (Sabin et al. 2010) and autophagy that would have opposing effects on viral success (McLean et al. 2011; Datan et al. 2016). The impact of the changes in expression seen here on ROS is not completely clear. Mitochondria produce more ROS when the NADH/NAD+ ratio increases (Murphy 2009). Less NADH dehydrogenase activity should therefore shift the balance in favor of ROS production. Less production of cytochrome subunits could also cause upstream components in the system to become loaded with electrons that in turn causes autoxidation, decreasing respiration, and increasing ROS (Ferguson et al. 2005). The effects of these particular changes on mosquito fitness are likely to be equally complex. For example, reduction in cytochrome C is known to be associated with shortened lifespan (Klichko et al. 2014) whereas less cytochrome B leads to lengthened lifespan (Copeland et al. 2009) in *D.melanogaster*.

This study has several constraints that should be noted. First, as expression was captured at a single time point, when virus arrived in saliva, we cannot speak to changeable patterns in expression that occurred prior and that may have contributed to EIP phenotypes. Second, while the dsRNA manipulations of ribosomal gene expression would have been telling about the functional effects on virus replication, we were unable to see an effect. It is possible that a large number of genes may need to be targeted simultaneously given the redundancy in ribosomal genes. Lastly, we used a rapid and sensitive qPCR-based method to quantify viral genome copy number in collected saliva samples and supernatants of infected cells (Hue et al. 2011). Plaque assays detect infectious particle but are not feasible for individual mosquitoes as saliva sample volumes are too small. Genome copy numbers represent an inflated, but closely correlated estimate of infectious virus particles (Ye, Chenoweth, et al. 2016) and are appropriate here given we were interested in relative comparisons of EIP rather than absolute values.

## Conclusions

The transcriptional profiling carried out here has allowed us to develop novel hypotheses about EIP and the rate of viral proliferation in mosquitoes, which require further testing in more manipulative studies. We suggest that the complex interplay between calcium ion distribution, ribosome biogenesis, and metabolism, possibly through shared connections in *mTor* may dictate the mosquito genetic component of EIP. The expression of these different pathways, that may vary naturally in the basal and induced states upon infection with DENV, also have energetic consequences for host fitness.

## Availability of Data

**All RNAseq Data Available in Figshare:** https://doi.org/10.26180/5bb0d5ae69035.

## Supplementary Material

Supplementary Data
