# Supplementary material for: The Transcriptional Response of Aedes aegypti with Variable Extrinsic Incubation Periods for Dengue Virus
Source: Genome Biol Evol. 2018 Oct 18;10(12):3141–51. doi: 10.1093/gbe/evy230 (PMC6278894; doi:10.1093/gbe/evy230)
Supplement: Supplementary Data [file evy230_supp.zip › Kohetalsupportinglegends.docx]

**Supp Figure 1. dsRNA knockdown in three candidate genes.** Expression of candidate genes (A) RpS9, (B) RpL9, and (C) RpL10 measured by qRT-PCR in Aag-2 cells at 2, 4, and 7 days post-transfection with dsRNA was compared to control mock-transfected cells. Expression levels shown were normalized to housekeeping gene, RpS17. Comparisons between dsRNA-treated and untreated cells were made using Student’s t-test or Mann-Whitney test (**, p < 0.01; ***, p < 0.001; ****, p < 0.0001). Mean and SEM are shown in graphs.

**Supp Figure 2. DENV titre following knockdown of candidate genes.** (A, B) Aag-2 cells were transfected with dsRNA targeting one of three candidate genes, followed by infection with DENV-3 at 1 day post-transfection (MOI = 0.01) over two separate experiments. Supernatants were collected from cells at 6 days post-infection. dsRNA-treated supernatants were compared to mock-treated control with Student’s t-test to find no significant differences in DENV copy number. Mean and SEM are shown in graphs.
