# Supplementary material for: The Transcriptional Response of Aedes aegypti with Variable Extrinsic Incubation Periods for Dengue Virus
Source: Genome Biol Evol. 2018 Oct 18;10(12):3141–51. doi: 10.1093/gbe/evy230 (PMC6278894; doi:10.1093/gbe/evy230)
Supplement: Supplementary Data [file evy230_supp.zip › Supp Table 1.docx]

**Supp Table 1. Enriched GO terms in genes significantly affected by dengue infection as a main effect**

| **GO term ID** | **GO term name** | **P-value** | **VectorBase Gene ID** |
| --- | --- | --- | --- |
| GO:0055114 | oxidation-reduction process | 2.49E-02 | AAEL002194,AAEL005790 |
| GO:0015985 | energy coupled proton transport, down electrochemical gradient | 3.69E-02 | AAEL002827,AAEL006256 |
| GO:0009144 | purine nucleoside triphosphate metabolic process | 4.82E-02 | AAEL002827,AAEL006256,AAEL018680 |
| GO:0009205 | purine ribonucleoside triphosphate metabolic process | 4.82E-02 | AAEL002827,AAEL006256,AAEL018680 |
| GO:0046034 | ATP metabolic process | 3.55E-02 | AAEL002827,AAEL006256,AAEL018680 |
| GO:0006754 | ATP biosynthetic process | 3.69E-02 | AAEL002827,AAEL006256 |
| GO:0015986 | ATP synthesis coupled proton transport | 3.69E-02 | AAEL002827,AAEL006256 |
| GO:0046653 | tetrahydrofolate metabolic process | 3.08E-02 | AAEL010764 |
| GO:0044281 | small molecule metabolic process | 1.30E-04 | AAEL000370, AAEL000454, AAEL001134, AAEL002194, AAEL002764, AAEL002827, AAEL004338, AAEL004613, AAEL005752, AAEL005790, AAEL006256, AAEL006347, AAEL007542, AAEL008963, AAEL009475, AAEL009490, AAEL010276, AAEL010737, AAEL010764, AAEL017098, AAEL018680 |
| GO:0055086 | nucleobase-containing small molecule metabolic process | 2.49E-02 | AAEL001134, AAEL002194, AAEL002827, AAEL006256, AAEL006347, AAEL009475, AAEL009490, AAEL018680 |
| GO:0008152 | metabolic process | 2.13E-02 | AAEL000037, AAEL000099, AAEL000128, AAEL000217, AAEL000343, AAEL000370, AAEL000427, AAEL000642, AAEL000834, AAEL001134, AAEL001312, AAEL001321, AAEL001566, AAEL001693, AAEL002194, AAEL002378, AAEL002565, AAEL002601, AAEL002721, AAEL002827, AAEL002892, AAEL003066, AAEL003071, AAEL003286, AAEL003349, AAEL003434, AAEL003642, AAEL003748, AAEL004338, AAEL004390, AAEL004613, AAEL005181, AAEL005453, AAEL005596, AAEL005645, AAEL005752, AAEL005790, AAEL006256, AAEL006347, AAEL006719, AAEL007397, AAEL007444, AAEL007541, AAEL007542, AAEL007653, AAEL007926, AAEL008097, AAEL008619, AAEL009061, AAEL009313, AAEL009637, AAEL009993, AAEL010196, AAEL010276, AAEL010513, AAEL010530, AAEL010596, AAEL010764, AAEL010776, AAEL010910, AAEL010935, AAEL011112, AAEL011756, AAEL011758, AAEL012750, AAEL013707, AAEL013714, AAEL013715, AAEL014188, AAEL014734, AAEL014797, AAEL017098, AAEL018159, AAEL018162, AAEL018680 |
| GO:0044238 | primary metabolic process | 2.72E-02 | AAEL000037, AAEL000059, AAEL000099, AAEL000128, AAEL000217, AAEL000343, AAEL000370, AAEL000454, AAEL000642, AAEL000654, AAEL001134, AAEL001321, AAEL001566, AAEL001693, AAEL002194, AAEL002565, AAEL002601, AAEL002721, AAEL002764, AAEL002827, AAEL002892, AAEL003066, AAEL003071, AAEL003434, AAEL003642, AAEL003699, AAEL004613, AAEL005181, AAEL005453, AAEL005521, AAEL005596, AAEL005645, AAEL005752, AAEL006256, AAEL006347, AAEL006674, AAEL006719, AAEL007397, AAEL007444, AAEL007541, AAEL007926, AAEL008097, AAEL008619, AAEL008963, AAEL009061, AAEL009313, AAEL009475, AAEL009490, AAEL009637, AAEL009992, AAEL009993, AAEL010196, AAEL010276, AAEL010513, AAEL010530, AAEL010596, AAEL010737, AAEL010776, AAEL010910, AAEL010912, AAEL011758, AAEL012750, AAEL013707, AAEL013714, AAEL013715, AAEL014188, AAEL017098, AAEL018159, AAEL018162, AAEL018680 |
| GO:0006807 | nitrogen compound metabolic process | 2.80E-02 | AAEL000037, AAEL000059, AAEL000099, AAEL000128, AAEL000217, AAEL000343, AAEL000654, AAEL001134, AAEL001321, AAEL001566, AAEL001693, AAEL002194, AAEL002565, AAEL002601, AAEL002721, AAEL002827, AAEL002892, AAEL003066, AAEL003071, AAEL003642, AAEL003699, AAEL004613, AAEL005181, AAEL005453, AAEL005521, AAEL005596, AAEL005645, AAEL006256, AAEL006347, AAEL006674, AAEL007397, AAEL007444, AAEL007541, AAEL007653, AAEL007926, AAEL008097, AAEL008619, AAEL008963, AAEL009061, AAEL009313, AAEL009475, AAEL009490, AAEL009637, AAEL009992, AAEL009993, AAEL010196, AAEL010276, AAEL010513, AAEL010530, AAEL010596, AAEL010737, AAEL010764, AAEL010776, AAEL010910, AAEL010912, AAEL010935, AAEL011758, AAEL012750, AAEL013707, AAEL013714, AAEL013715, AAEL014188, AAEL017098, AAEL018159, AAEL018162, AAEL018680 |
| GO:0071704 | organic substance metabolic process | 1.44E-02 | AAEL000037, AAEL000059, AAEL000099, AAEL000128, AAEL000217, AAEL000343, AAEL000370, AAEL000454, AAEL000642, AAEL000654, AAEL001134, AAEL001321, AAEL001566, AAEL001693, AAEL002194, AAEL002565, AAEL002601, AAEL002721, AAEL002764, AAEL002827, AAEL002892, AAEL003066, AAEL003071, AAEL003434, AAEL003642, AAEL003699, AAEL004338, AAEL004613, AAEL005181, AAEL005453, AAEL005521, AAEL005596, AAEL005645, AAEL005752, AAEL005790, AAEL006256, AAEL006347, AAEL006674, AAEL006719, AAEL007397, AAEL007444, AAEL007541, AAEL007542, AAEL007653, AAEL007926, AAEL008097, AAEL008619, AAEL008963, AAEL009061, AAEL009313, AAEL009475, AAEL009490, AAEL009637, AAEL009992, AAEL009993, AAEL010196, AAEL010276, AAEL010513, AAEL010530, AAEL010596, AAEL010737, AAEL010764, AAEL010776, AAEL010910, AAEL010912, AAEL010935, AAEL011758, AAEL012750, AAEL013707, AAEL013714, AAEL013715, AAEL014188, AAEL017098, AAEL018159, AAEL018162, AAEL018680 |
| GO:1901564 | organonitrogen compound metabolic process | 8.29E-04 | AAEL000037, AAEL000059, AAEL000099, AAEL000217, AAEL000343, AAEL000654, AAEL001134, AAEL001566, AAEL001693, AAEL002194, AAEL002565, AAEL002601, AAEL002827, AAEL002892, AAEL003066, AAEL003642, AAEL003699, AAEL004613, AAEL005181, AAEL005521, AAEL005596, AAEL005645, AAEL006256, AAEL006674, AAEL007653, AAEL007926, AAEL008097, AAEL008619, AAEL008963, AAEL009313, AAEL009475, AAEL009490, AAEL009637, AAEL009992, AAEL009993, AAEL010196, AAEL010276, AAEL010530, AAEL010596, AAEL010737, AAEL010764, AAEL010776, AAEL010910, AAEL010912, AAEL010935, AAEL011758, AAEL013707, AAEL013714, AAEL013715, AAEL014188, AAEL017098, AAEL018159, AAEL018162, AAEL018680 |
| GO:0009112 | nucleobase metabolic process | 1.13E-04 | AAEL001134, AAEL002194, AAEL009475, AAEL009490 |
| GO:0046112 | nucleobase biosynthetic process | 4.68E-02 | AAEL009475, AAEL009490 |
| GO:1901565 | organonitrogen compound catabolic process | 1.44E-02 | AAEL002194, AAEL007653, AAEL010764 |
| GO:0042219 | cellular modified amino acid catabolic process | 3.54E-02 | AAEL010764, AAEL010935 |
| GO:0019538 | protein metabolic process | 4.07E-02 | AAEL000037, AAEL000059, AAEL000099, AAEL000217, AAEL000343, AAEL000654, AAEL001566, AAEL001693, AAEL002565, AAEL002601, AAEL002892, AAEL003642, AAEL003699, AAEL004613, AAEL005181, AAEL005521, AAEL005596, AAEL005645, AAEL006674, AAEL007926, AAEL008097, AAEL008619, AAEL009313, AAEL009637, AAEL009992, AAEL009993, AAEL010196, AAEL010530, AAEL010596, AAEL010776, AAEL010910, AAEL010912, AAEL011758, AAEL013707, AAEL013714, AAEL013715, AAEL014188, AAEL018159, AAEL018162 |
| GO:0006508 | proteolysis | 2.26E-03 | AAEL000037, AAEL000059, AAEL000099, AAEL001566, AAEL001693, AAEL002601, AAEL003642, AAEL003699, AAEL005521, AAEL005596, AAEL005645, AAEL006674, AAEL007926, AAEL008097, AAEL008619, AAEL009637, AAEL009992, AAEL009993, AAEL010196, AAEL010776, AAEL010912, AAEL013707, AAEL013714, AAEL013715, AAEL014188, AAEL018159 |
| GO:0097264 | self proteolysis | 3.79E-04 | AAEL003699, AAEL009992, AAEL009993 |
| GO:0044712 | single-organism catabolic process | 4.64E-02 | AAEL000370, AAEL002194, AAEL006347, AAEL010276, AAEL010764 |
| GO:0044282 | small molecule catabolic process | 3.06E-03 | AAEL000370, AAEL002194, AAEL010276, AAEL010764 |
| GO:1901361 | organic cyclic compound catabolic process | 2.47E-04 | AAEL002194, AAEL006347, AAEL007653, AAEL010764 |
| GO:0044248 | cellular catabolic process | 3.17E-02 | AAEL002194, AAEL007653, AAEL010764 |
| GO:0044270 | cellular nitrogen compound catabolic process | 1.95E-04 | AAEL002194, AAEL006347, AAEL007653, AAEL010764 |
| GO:0043605 | cellular amide catabolic process | 2.64E-02 | AAEL007653 |
| GO:0046700 | heterocycle catabolic process | 2.11E-04 | AAEL002194, AAEL006347, AAEL007653, AAEL010764 |
| GO:0000256 | allantoin catabolic process | 2.64E-02 | AAEL007653 |
| GO:0009109 | coenzyme catabolic process | 1.03E-02 | AAEL010764 |
| GO:0072527 | pyrimidine-containing compound metabolic process | 1.79E-02 | AAEL001134, AAEL009475, AAEL009490 |
| GO:0006206 | pyrimidine nucleobase metabolic process | 4.99E-04 | AAEL001134, AAEL009475, AAEL009490 |
| GO:0019856 | pyrimidine nucleobase biosynthetic process | 1.72E-02 | AAEL009475, AAEL009490 |
| GO:0006207 | de novo' pyrimidine nucleobase biosynthetic process | 1.72E-02 | AAEL009475, AAEL009490 |
| GO:0006082 | organic acid metabolic process | 2.74E-05 | AAEL000370, AAEL000454, AAEL001134, AAEL002194, AAEL002764, AAEL004338, AAEL004613, AAEL005790, AAEL007542, AAEL008963, AAEL009475, AAEL009490, AAEL010276, AAEL010737, AAEL010764, AAEL017098 |
| GO:0009109 | coenzyme catabolic process | 1.03E-02 | AAEL010764 |
| GO:0043436 | oxoacid metabolic process | 1.16E-04 | AAEL000370, AAEL000454, AAEL001134, AAEL002764, AAEL004338, AAEL004613, AAEL005790, AAEL007542, AAEL008963, AAEL009475, AAEL009490, AAEL010276, AAEL010737, AAEL010764, AAEL017098 |
| GO:0019752 | carboxylic acid metabolic process | 9.57E-05 | AAEL000370, AAEL000454, AAEL001134, AAEL002764, AAEL004338, AAEL004613, AAEL005790, AAEL007542, AAEL008963, AAEL009475, AAEL009490, AAEL010276, AAEL010737, AAEL010764, AAEL017098 |
| GO:0043648 | dicarboxylic acid metabolic process | 7.82E-04 | AAEL005790, AAEL010764 |
| GO:0009256 | 10-formyltetrahydrofolate metabolic process | 1.03E-02 | AAEL010764 |
| GO:0006108 | malate metabolic process | 2.94E-03 | AAEL005790 |
| GO:0006520 | cellular amino acid metabolic process | 9.13E-03 | AAEL001134, AAEL004613, AAEL008963, AAEL009475, AAEL009490, AAEL010276, AAEL010737, AAEL017098 |
| GO:0016054 | organic acid catabolic process | 1.65E-02 | AAEL000370, AAEL010276, AAEL010764 |
| GO:0046395 | carboxylic acid catabolic process | 1.65E-02 | AAEL000370, AAEL010276, AAEL010764 |
| GO:0043649 | dicarboxylic acid catabolic process | 1.03E-02 | AAEL010764 |
| GO:0072521 | purine-containing compound metabolic process | 3.13E-02 | AAEL002194, AAEL002827, AAEL006256, AAEL018680 |
| GO:0046415 | urate metabolic process | 1.47E-03 | AAEL002194 |
| GO:0006144 | purine nucleobase metabolic process | 7.35E-03 | AAEL002194 |
| GO:0019439 | aromatic compound catabolic process | 5.95E-03 | AAEL002194, AAEL006347, AAEL010764 |
| GO:0072523 | purine-containing compound catabolic process | 4.41E-03 | AAEL002194 |
| GO:0019628 | urate catabolic process | 1.47E-03 | AAEL002194 |
| GO:0042560 | pteridine-containing compound catabolic process | 1.03E-02 | AAEL010764 |
| GO:0009397 | folic acid-containing compound catabolic process | 1.03E-02 | AAEL010764 |
| GO:0009258 | 10-formyltetrahydrofolate catabolic process | 1.03E-02 | AAEL010764 |
