# Supplementary material for: The Transcriptional Response of Aedes aegypti with Variable Extrinsic Incubation Periods for Dengue Virus
Source: Genome Biol Evol. 2018 Oct 18;10(12):3141–51. doi: 10.1093/gbe/evy230 (PMC6278894; doi:10.1093/gbe/evy230)
Supplement: Supplementary Data [file evy230_supp.zip › Supp Table 2.docx]

**Supp Table 2. Enriched GO terms in genes significantly affected by the interaction of EIP and infection.**

GO terms in bold showed interesting expression pattern across the four EIP phenotypes and are the subject of detailed discussion in this paper.

| **GO term ID** | **GO term name** | **P-value** | **VectorBase Gene ID** |
| --- | --- | --- | --- |
| GO:0019693 | ribose phosphate metabolic process | 4.84E-02 | AAEL001668, AAEL009389, AAEL018658, AAEL018668, AAEL018685 |
| GO:0030155 | regulation of cell adhesion | 4.56E-02 | AAEL005667 |
| GO:0010810 | regulation of cell-substrate adhesion | 4.56E-02 | AAEL005667 |
| GO:0001952 | regulation of cell-matrix adhesion | 4.56E-02 | AAEL005667 |
| GO:0009607 | response to biotic stimulus | 1.19E-02 | AAEL000611, AAEL003841 |
| GO:0009605 | response to external stimulus | 7.57E-04 | AAEL000611, AAEL003841, AAEL006259, AAEL009615 |
| GO:0043207 | response to external biotic stimulus | 1.19E-02 | AAEL000611, AAEL003841 |
| GO:0051707 | response to other organism | 1.19E-02 | AAEL000611, AAEL003841 |
| GO:0009617 | response to bacterium | 1.19E-02 | AAEL000611, AAEL003841 |
| GO:0009628 | response to abiotic stimulus | 3.03E-02 | AAEL009615 |
| GO:0009314 | response to radiation | 2.09E-02 | AAEL009615 |
| GO:0009416 | response to light stimulus | 2.09E-02 | AAEL009615 |
| GO:0051606 | detection of stimulus | 3.60E-02 | AAEL009615 |
| GO:0009582 | detection of abiotic stimulus | 1.53E-02 | AAEL006259, AAEL009615 |
| GO:0009581 | detection of external stimulus | 1.53E-02 | AAEL006259, AAEL009615 |
| GO:0009583 | detection of light stimulus | 1.53E-02 | AAEL006259, AAEL009615 |
| GO:0007602 | phototransduction | 1.53E-02 | AAEL006259, AAEL009615 |
| **GO:0006952** | **defense response** | **4.14E-03** | **AAEL000611, AAEL003841, AAEL003857** |
| GO:0098542 | defense response to other organism | 1.19E-02 | AAEL000611, AAEL003841 |
| GO:0042742 | defense response to bacterium | 1.19E-02 | AAEL000611, AAEL003841 |
| GO:0050830 | defense response to Gram-positive bacterium | 3.12E-02 | AAEL003841 |
| GO:0050829 | defense response to Gram-negative bacterium | 3.12E-02 | AAEL003841 |
| GO:0050953 | sensory perception of light stimulus | 2.84E-02 | AAEL009615 |
| GO:0007601 | visual perception | 2.47E-02 | AAEL009615 |
| GO:0018023 | peptidyl-lysine trimethylation | 2.24E-02 | AAEL008027 |
| GO:0034770 | histone H4-K20 methylation | 2.24E-02 | AAEL008027 |
| GO:0034773 | histone H4-K20 trimethylation | 2.24E-02 | AAEL008027 |
| GO:0007186 | G-protein coupled receptor signaling pathway | 1.31E-02 | AAEL009024, AAEL009615 |
| GO:0022613 | ribonucleoprotein complex biogenesis | 1.71E-02 | AAEL003352, AAEL005722, AAEL010821 |
| GO:0042254 | ribosome biogenesis | 8.51E-03 | AAEL003352, AAEL005722, AAEL010821 |
| **GO:0070588** | **calcium ion transmembrane transport** | **3.64E-02** | **AAEL003837, AAEL006582** |
| GO:0009141 | nucleoside triphosphate metabolic process | 4.29E-02 | AAEL001668, AAEL018658, AAEL018668, AAEL018685 |
| GO:0009144 | purine nucleoside triphosphate metabolic process | 3.40E-02 | AAEL001668, AAEL018658, AAEL018668, AAEL018685 |
| GO:0009199 | ribonucleoside triphosphate metabolic process | 3.61E-02 | AAEL001668, AAEL018658, AAEL018668, AAEL018685 |
| GO:0009205 | purine ribonucleoside triphosphate metabolic process | 3.40E-02 | AAEL001668, AAEL018658, AAEL018668, AAEL018685 |
| GO:0008150 | biological_process | 1.49E-04 | AAEL000010, AAEL000471, AAEL000611, AAEL000670, AAEL000823, AAEL000951, AAEL000987, AAEL001091, AAEL001324, AAEL001504, AAEL001668, AAEL001759, AAEL002372, AAEL002467, AAEL002534, AAEL002582, AAEL002781, AAEL003116, AAEL003352, AAEL003399, AAEL003433, AAEL003582, AAEL003837, AAEL003841, AAEL003857, AAEL003873, AAEL003942, AAEL004151, AAEL004175, AAEL004503, AAEL004542, AAEL005085, AAEL005097, AAEL005266, AAEL005451, AAEL005480, AAEL005629, AAEL005667, AAEL005722, AAEL005748, AAEL005793, AAEL005802, AAEL005817, AAEL005901, AAEL006259, AAEL006511, AAEL006518, AAEL006568, AAEL006576, AAEL006582, AAEL006721, AAEL006785, AAEL006860, AAEL007022, AAEL007042, AAEL007254, AAEL007699, AAEL007728, AAEL007842, AAEL008027, AAEL008083, AAEL008123, AAEL008141, AAEL008192, AAEL008345, AAEL008953, AAEL009024, AAEL009151, AAEL009362, AAEL009389, AAEL009496, AAEL009524, AAEL009615, AAEL009658, AAEL009747, AAEL009955, AAEL009994, AAEL010047, AAEL010145, AAEL010168, AAEL010821, AAEL010884, AAEL011184, AAEL011371, AAEL011447, AAEL011471, AAEL011656, AAEL012098, AAEL012733, AAEL012944, AAEL013221, AAEL013272, AAEL013535, AAEL014238, AAEL014494, AAEL014562, AAEL014897, AAEL015523, AAEL017096, AAEL017516, AAEL018057, AAEL018658, AAEL018662, AAEL018664, AAEL018668, AAEL018669, AAEL018671, AAEL018685, AAEL018687 |
| GO:0008152 | metabolic process | 5.33E-06 | AAEL000010, AAEL000670, AAEL000823, AAEL000951, AAEL000987, AAEL001091, AAEL001324, AAEL001504, AAEL001668, AAEL001759, AAEL002372, AAEL002467, AAEL002534, AAEL002582, AAEL002781, AAEL003399, AAEL003582, AAEL003873, AAEL003942, AAEL004151, AAEL004175, AAEL004503, AAEL004542, AAEL005085, AAEL005097, AAEL005266, AAEL005451, AAEL005480, AAEL005629, AAEL005748, AAEL005793, AAEL005802, AAEL005817, AAEL005901, AAEL006259, AAEL006511, AAEL006518, AAEL006568, AAEL006576, AAEL006721, AAEL006785, AAEL006860, AAEL007022, AAEL007042, AAEL007254, AAEL007699, AAEL008027, AAEL008083, AAEL008123, AAEL008141, AAEL008192, AAEL008345, AAEL008953, AAEL009151, AAEL009389, AAEL009496, AAEL009524, AAEL009658, AAEL009747, AAEL009994, AAEL010168, AAEL011371, AAEL011447, AAEL011471, AAEL011656, AAEL012733, AAEL012944, AAEL013221, AAEL013272, AAEL014238, AAEL014494, AAEL014562, AAEL014897, AAEL015523, AAEL017096, AAEL017516, AAEL018057, AAEL018658, AAEL018662, AAEL018664, AAEL018668, AAEL018669, AAEL018671, AAEL018685, AAEL018687 |
| GO:0044238 | primary metabolic process | 1.08E-06 | AAEL000010, AAEL000823, AAEL000951, AAEL000987, AAEL001324, AAEL001504, AAEL001668, AAEL001759, AAEL002372, AAEL002534, AAEL002582, AAEL002781, AAEL003582, AAEL003942, AAEL004151, AAEL004175, AAEL004503, AAEL005085, AAEL005097, AAEL005266, AAEL005451, AAEL005480, AAEL005629, AAEL005748, AAEL005802, AAEL005817, AAEL005901, AAEL006259, AAEL006511, AAEL006518, AAEL006568, AAEL006576, AAEL006721, AAEL006785, AAEL006860, AAEL007022, AAEL007042, AAEL007254, AAEL007699, AAEL008027, AAEL008083, AAEL008123, AAEL008141, AAEL008192, AAEL008953, AAEL009151, AAEL009389, AAEL009496, AAEL009524, AAEL009658, AAEL009747, AAEL009994, AAEL010168, AAEL011371, AAEL011447, AAEL011471, AAEL011656, AAEL012733, AAEL012944, AAEL013221, AAEL013272, AAEL014238, AAEL014494, AAEL014562, AAEL014897, AAEL015523, AAEL017096, AAEL017516, AAEL018057, AAEL018658, AAEL018668, AAEL018685 |
| GO:0009058 | biosynthetic process | 3.44E-12 | AAEL000010, AAEL000823, AAEL000951, AAEL000987, AAEL001324, AAEL001759, AAEL002534, AAEL003582, AAEL003942, AAEL004151, AAEL004503, AAEL005085, AAEL005097, AAEL005266, AAEL005451, AAEL005480, AAEL005629, AAEL005817, AAEL005901, AAEL006511, AAEL006785, AAEL006860, AAEL007022, AAEL007042, AAEL007699, AAEL008141, AAEL008192, AAEL008953, AAEL009151, AAEL009496, AAEL009994, AAEL010168, AAEL011371, AAEL011447, AAEL011471, AAEL011656, AAEL012733, AAEL012944, AAEL013221, AAEL013272, AAEL014494, AAEL014562, AAEL017096, AAEL018668 |
| GO:0071704 | organic substance metabolic process | 5.62E-06 | AAEL000010, AAEL000823, AAEL000951, AAEL000987, AAEL001324, AAEL001504, AAEL001668, AAEL001759, AAEL002372, AAEL002467, AAEL002534, AAEL002582, AAEL002781, AAEL003582, AAEL003873, AAEL003942, AAEL004151, AAEL004175, AAEL004503, AAEL005085, AAEL005097, AAEL005266, AAEL005451, AAEL005480, AAEL005629, AAEL005748, AAEL005802, AAEL005817, AAEL005901, AAEL006259, AAEL006511, AAEL006518, AAEL006568, AAEL006576, AAEL006721, AAEL006785, AAEL006860, AAEL007022, AAEL007042, AAEL007254, AAEL007699, AAEL008027, AAEL008083, AAEL008123, AAEL008141, AAEL008192, AAEL008953, AAEL009151, AAEL009389, AAEL009496, AAEL009524, AAEL009658, AAEL009747, AAEL009994, AAEL010168, AAEL011371, AAEL011447, AAEL011471, AAEL011656, AAEL012733, AAEL012944, AAEL013221, AAEL013272, AAEL014238, AAEL014494, AAEL014562, AAEL014897, AAEL015523, AAEL017096, AAEL017516, AAEL018057, AAEL018658, AAEL018668, AAEL018685 |
| GO:0043170 | macromolecule metabolic process | 2.58E-06 | AAEL000010, AAEL000823, AAEL000951, AAEL000987, AAEL001324, AAEL001504, AAEL001759, AAEL002534, AAEL003582, AAEL003942, AAEL004151, AAEL004503, AAEL005085, AAEL005097, AAEL005266, AAEL005451, AAEL005480, AAEL005629, AAEL005802, AAEL005817, AAEL005901, AAEL006259, AAEL006511, AAEL006568, AAEL006576, AAEL006785, AAEL006860, AAEL007022, AAEL007042, AAEL007254, AAEL007699, AAEL008027, AAEL008123, AAEL008141, AAEL008192, AAEL008953, AAEL009151, AAEL009496, AAEL009994, AAEL010168, AAEL011371, AAEL011447, AAEL011471, AAEL011656, AAEL012733, AAEL012944, AAEL013221, AAEL013272, AAEL014494, AAEL014562, AAEL015523, AAEL017096, AAEL018057 |
| GO:0010467 | gene expression | 3.68E-14 | AAEL000010, AAEL000823, AAEL000951, AAEL000987, AAEL001759, AAEL002372, AAEL002534, AAEL003582, AAEL003942, AAEL004151, AAEL004175, AAEL004503, AAEL005085, AAEL005097, AAEL005266, AAEL005451, AAEL005480, AAEL005629, AAEL005817, AAEL005901, AAEL006511, AAEL006785, AAEL006860, AAEL007022, AAEL007042, AAEL007699, AAEL008083, AAEL008141, AAEL008192, AAEL008953, AAEL009151, AAEL009496, AAEL009994, AAEL010168, AAEL011371, AAEL011447, AAEL011471, AAEL011656, AAEL012733, AAEL012944, AAEL013221, AAEL013272, AAEL014494, AAEL014562, AAEL017096, AAEL017516 |
| GO:1901576 | organic substance biosynthetic process | 1.12E-12 | AAEL000010, AAEL000823, AAEL000951, AAEL000987, AAEL001324, AAEL001759, AAEL002534, AAEL003582, AAEL003942, AAEL004151, AAEL004503, AAEL005085, AAEL005097, AAEL005266, AAEL005451, AAEL005480, AAEL005629, AAEL005817, AAEL005901, AAEL006511, AAEL006785, AAEL006860, AAEL007022, AAEL007042, AAEL007699, AAEL008141, AAEL008192, AAEL008953, AAEL009151, AAEL009496, AAEL009994, AAEL010168, AAEL011371, AAEL011447, AAEL011471, AAEL011656, AAEL012733, AAEL012944, AAEL013221, AAEL013272, AAEL014494, AAEL014562, AAEL017096, AAEL018668 |
| GO:0009059 | macromolecule biosynthetic process | 1.52E-16 | AAEL000010, AAEL000823, AAEL000951, AAEL000987, AAEL001324, AAEL001759, AAEL002372, AAEL002534, AAEL003582, AAEL003942, AAEL004151, AAEL004175, AAEL004503, AAEL005085, AAEL005097, AAEL005266, AAEL005451, AAEL005480, AAEL005629, AAEL005817, AAEL005901, AAEL006511, AAEL006785, AAEL006860, AAEL007022, AAEL007042, AAEL007699, AAEL008083, AAEL008141, AAEL008192, AAEL008953, AAEL009151, AAEL009496, AAEL009994, AAEL010168, AAEL011371, AAEL011447, AAEL011471, AAEL011656, AAEL012733, AAEL012944, AAEL013221, AAEL013272, AAEL014494, AAEL014562, AAEL017096, AAEL017516 |
| GO:0006807 | nitrogen compound metabolic process | 7.68E-07 | AAEL000010, AAEL000823, AAEL000951, AAEL000987, AAEL001324, AAEL001504, AAEL001668, AAEL001759, AAEL002372, AAEL002467, AAEL002534, AAEL002582, AAEL003582, AAEL003942, AAEL004151, AAEL004175, AAEL004503, AAEL005085, AAEL005097, AAEL005266, AAEL005451, AAEL005480, AAEL005629, AAEL005748, AAEL005802, AAEL005817, AAEL005901, AAEL006259, AAEL006511, AAEL006518, AAEL006568, AAEL006576, AAEL006785, AAEL006860, AAEL007022, AAEL007042, AAEL007254, AAEL007699, AAEL008027, AAEL008083, AAEL008123, AAEL008141, AAEL008192, AAEL008953, AAEL009151, AAEL009389, AAEL009496, AAEL009747, AAEL009994, AAEL010168, AAEL011371, AAEL011447, AAEL011471, AAEL011656, AAEL012733, AAEL012944, AAEL013221, AAEL013272, AAEL014238, AAEL014494, AAEL014562, AAEL014897, AAEL015523, AAEL017096, AAEL017516, AAEL018057, AAEL018658, AAEL018668, AAEL018685 |
| GO:1901564 | organonitrogen compound metabolic process | 5.92E-10 | AAEL000010, AAEL000823, AAEL000951, AAEL000987, AAEL001504, AAEL001668, AAEL001759, AAEL002372, AAEL002467, AAEL002534, AAEL002582, AAEL003582, AAEL003942, AAEL004151, AAEL004175, AAEL004503, AAEL005085, AAEL005097, AAEL005266, AAEL005451, AAEL005629, AAEL005748, AAEL005817, AAEL005901, AAEL006259, AAEL006511, AAEL006518, AAEL006568, AAEL006576, AAEL006785, AAEL006860, AAEL007254, AAEL007699, AAEL008027, AAEL008083, AAEL008192, AAEL009151, AAEL009389, AAEL009496, AAEL009747, AAEL009994, AAEL010168, AAEL011447, AAEL011471, AAEL011656, AAEL012733, AAEL012944, AAEL013221, AAEL013272, AAEL014238, AAEL014494, AAEL014562, AAEL014897, AAEL015523, AAEL017096, AAEL017516, AAEL018057, AAEL018658, AAEL018668, AAEL018685 |
| GO:1901566 | organonitrogen compound biosynthetic process | 1.57E-22 | AAEL000010, AAEL000823, AAEL000951, AAEL000987, AAEL001759, AAEL002372, AAEL002534, AAEL003582, AAEL003942, AAEL004151, AAEL004175, AAEL004503, AAEL005085, AAEL005097, AAEL005266, AAEL005451, AAEL005629, AAEL005817, AAEL005901, AAEL006511, AAEL006785, AAEL006860, AAEL007699, AAEL008083, AAEL008192, AAEL009151, AAEL009496, AAEL009994, AAEL010168, AAEL011447, AAEL011471, AAEL011656, AAEL012733, AAEL012944, AAEL013221, AAEL013272, AAEL014494, AAEL014562, AAEL017096, AAEL017516, AAEL018668 |
| GO:0019538 | protein metabolic process | 3.35E-10 | AAEL000010, AAEL000823, AAEL000951, AAEL000987, AAEL001504, AAEL001759, AAEL002372, AAEL002534, AAEL002582, AAEL003582, AAEL003942, AAEL004151, AAEL004175, AAEL004503, AAEL005085, AAEL005097, AAEL005266, AAEL005451, AAEL005629, AAEL005748, AAEL005817, AAEL005901, AAEL006259, AAEL006511, AAEL006568, AAEL006576, AAEL006785, AAEL006860, AAEL007254, AAEL007699, AAEL008027, AAEL008083, AAEL008192, AAEL009151, AAEL009496, AAEL009747, AAEL009994, AAEL010168, AAEL011447, AAEL011471, AAEL011656, AAEL012733, AAEL012944, AAEL013221, AAEL013272, AAEL014494, AAEL014562, AAEL014897, AAEL015523, AAEL017096, AAEL017516, AAEL018057 |
| GO:0009987 | cellular process | 0.0000005 | AAEL000010, AAEL000823, AAEL000951, AAEL000987, AAEL001324, AAEL001504, AAEL001668, AAEL001759, AAEL002372, AAEL002534, AAEL002781, AAEL003116, AAEL003582, AAEL003837, AAEL003873, AAEL003942, AAEL004151, AAEL004175, AAEL004503, AAEL005085, AAEL005097, AAEL005266, AAEL005451, AAEL005480, AAEL005629, AAEL005802, AAEL005817, AAEL005901, AAEL006259, AAEL006511, AAEL006518, AAEL006721, AAEL006785, AAEL006860, AAEL007022, AAEL007042, AAEL007699, AAEL007728, AAEL007842, AAEL008027, AAEL008083, AAEL008123, AAEL008141, AAEL008192, AAEL008953, AAEL009024, AAEL009151, AAEL009389, AAEL009496, AAEL009615, AAEL009658, AAEL009994, AAEL010168, AAEL011371, AAEL011447, AAEL011471, AAEL011656, AAEL012733, AAEL012944, AAEL013221, AAEL013272, AAEL013535, AAEL014238, AAEL014494, AAEL014562, AAEL015523, AAEL017096, AAEL017516, AAEL018057, AAEL018658, AAEL018662, AAEL018664, AAEL018668, AAEL018669 |
| GO:0044237 | cellular metabolic process | 8.49E-11 | AAEL000010, AAEL000823, AAEL000951, AAEL000987, AAEL001324, AAEL001504, AAEL001668, AAEL001759, AAEL002372, AAEL002534, AAEL002781, AAEL003582, AAEL003873, AAEL003942, AAEL004151, AAEL004175, AAEL004503, AAEL005085, AAEL005097, AAEL005266, AAEL005451, AAEL005480, AAEL005629, AAEL005802, AAEL005817, AAEL005901, AAEL006259, AAEL006511, AAEL006518, AAEL006721, AAEL006785, AAEL006860, AAEL007022, AAEL007042, AAEL007699, AAEL008027, AAEL008083, AAEL008123, AAEL008141, AAEL008192, AAEL008953, AAEL009151, AAEL009389, AAEL009496, AAEL009658, AAEL009747, AAEL009994, AAEL010168, AAEL011371, AAEL011447, AAEL011471, AAEL011656, AAEL012733, AAEL012944, AAEL013221, AAEL013272, AAEL014238, AAEL014494, AAEL014562, AAEL015523, AAEL017096, AAEL017516, AAEL018057, AAEL018658, AAEL018662, AAEL018664, AAEL018668, AAEL018669, AAEL018685 |
| GO:0034641 | cellular nitrogen compound metabolic process | 7.5E-14 | AAEL000010, AAEL000823, AAEL000951, AAEL000987, AAEL001759, AAEL002372, AAEL002534, AAEL003582, AAEL003942, AAEL004151, AAEL004175, AAEL004503, AAEL005085, AAEL005097, AAEL005266, AAEL005451, AAEL005629, AAEL005817, AAEL005901, AAEL006511, AAEL006785, AAEL006860, AAEL007699, AAEL008083, AAEL008192, AAEL009151, AAEL009496, AAEL009747, AAEL009994, AAEL010168, AAEL011447, AAEL011471, AAEL011656, AAEL012733, AAEL012944, AAEL013221, AAEL013272, AAEL014494, AAEL014562, AAEL017096, AAEL017516 |
| GO:0043603 | cellular amide metabolic process | 8.5E-30 | AAEL000010, AAEL000823, AAEL000951, AAEL000987, AAEL001759, AAEL002372, AAEL002534, AAEL003582, AAEL003942, AAEL004151, AAEL004175, AAEL004503, AAEL005085, AAEL005097, AAEL005266, AAEL005451, AAEL005629, AAEL005817, AAEL005901, AAEL006511, AAEL006785, AAEL006860, AAEL007699, AAEL008083, AAEL008192, AAEL009151, AAEL009496, AAEL009747, AAEL009994, AAEL010168, AAEL011447, AAEL011471, AAEL011656, AAEL012733, AAEL012944, AAEL013221, AAEL013272, AAEL014494, AAEL014562, AAEL017096, AAEL017516 |
| GO:0006518 | peptide metabolic process | 7.66E-32 | AAEL000010, AAEL000823, AAEL000951, AAEL000987, AAEL001759, AAEL002372, AAEL002534, AAEL003582, AAEL003942, AAEL004151, AAEL004175, AAEL004503, AAEL005085, AAEL005097, AAEL005266, AAEL005451, AAEL005629, AAEL005817, AAEL005901, AAEL006511, AAEL006785, AAEL006860, AAEL007699, AAEL008083, AAEL008192, AAEL009151, AAEL009496, AAEL009747, AAEL009994, AAEL010168, AAEL011447, AAEL011471, AAEL011656, AAEL012733, AAEL012944, AAEL013221, AAEL013272, AAEL014494, AAEL014562, AAEL017096, AAEL017516 |
| GO:0044260 | cellular macromolecule metabolic process | 1.03E-10 | AAEL000010, AAEL000823, AAEL000951, AAEL000987, AAEL001324, AAEL001504, AAEL001759, AAEL002534, AAEL003582, AAEL003942, AAEL004151, AAEL004503, AAEL005085, AAEL005097, AAEL005266, AAEL005451, AAEL005480, AAEL005629, AAEL005802, AAEL005817, AAEL005901, AAEL006259, AAEL006511, AAEL006785, AAEL006860, AAEL007022, AAEL007042, AAEL007699, AAEL008027, AAEL008123, AAEL008141, AAEL008192, AAEL008953, AAEL009151, AAEL009496, AAEL009994, AAEL010168, AAEL011371, AAEL011447, AAEL011471, AAEL011656, AAEL012733, AAEL012944, AAEL013221, AAEL013272, AAEL014494, AAEL014562, AAEL015523, AAEL017096, AAEL018057 |
| GO:0044267 | cellular protein metabolic process | 4.27E-16 | AAEL000010, AAEL000823, AAEL000951, AAEL000987, AAEL001504, AAEL001759, AAEL002372, AAEL002534, AAEL003582, AAEL003942, AAEL004151, AAEL004175, AAEL004503, AAEL005085, AAEL005097, AAEL005266, AAEL005451, AAEL005629, AAEL005817, AAEL005901, AAEL006259, AAEL006511, AAEL006785, AAEL006860, AAEL007699, AAEL008027, AAEL008083, AAEL008192, AAEL009151, AAEL009496, AAEL009994, AAEL010168, AAEL011447, AAEL011471, AAEL011656, AAEL012733, AAEL012944, AAEL013221, AAEL013272, AAEL014494, AAEL014562, AAEL015523, AAEL017096, AAEL017516, AAEL018057 |
| GO:0006091 | generation of precursor metabolites and energy | 3.25E-04 | AAEL001668, AAEL006721, AAEL018658, AAEL018662, AAEL018664, AAEL018669, AAEL018685 |
| GO:0044249 | cellular biosynthetic process | 6.84E-13 | AAEL000010, AAEL000823, AAEL000951, AAEL000987, AAEL001324, AAEL001759, AAEL002534, AAEL003582, AAEL003942, AAEL004151, AAEL004503, AAEL005085, AAEL005097, AAEL005266, AAEL005451, AAEL005480, AAEL005629, AAEL005817, AAEL005901, AAEL006511, AAEL006785, AAEL006860, AAEL007022, AAEL007042, AAEL007699, AAEL008141, AAEL008192, AAEL008953, AAEL009151, AAEL009496, AAEL009994, AAEL010168, AAEL011371, AAEL011447, AAEL011471, AAEL011656, AAEL012733, AAEL012944, AAEL013221, AAEL013272, AAEL014494, AAEL014562, AAEL017096, AAEL018668 |
| GO:0044271 | cellular nitrogen compound biosynthetic process | 1.40E-16 | AAEL000010, AAEL000823, AAEL000951, AAEL000987, AAEL001759, AAEL002372, AAEL002534, AAEL003582, AAEL003942, AAEL004151, AAEL004175, AAEL004503, AAEL005085, AAEL005097, AAEL005266, AAEL005451, AAEL005480, AAEL005629, AAEL005817, AAEL005901, AAEL006511, AAEL006785, AAEL006860, AAEL007022, AAEL007042, AAEL007699, AAEL008083, AAEL008141, AAEL008192, AAEL008953, AAEL009151, AAEL009496, AAEL009994, AAEL010168, AAEL011371, AAEL011447, AAEL011471, AAEL011656, AAEL012733, AAEL012944, AAEL013221, AAEL013272, AAEL014494, AAEL014562, AAEL017096, AAEL017516, AAEL018668 |
| GO:0043604 | amide biosynthetic process | 3.46E-32 | AAEL000010, AAEL000823, AAEL000951, AAEL000987, AAEL001759, AAEL002372, AAEL002534, AAEL003582, AAEL003942, AAEL004151, AAEL004175, AAEL004503, AAEL005085, AAEL005097, AAEL005266, AAEL005451, AAEL005629, AAEL005817, AAEL005901, AAEL006511, AAEL006785, AAEL006860, AAEL007699, AAEL008083, AAEL008192, AAEL009151, AAEL009496, AAEL009747, AAEL009994, AAEL010168, AAEL011447, AAEL011471, AAEL011656, AAEL012733, AAEL012944, AAEL013221, AAEL013272, AAEL014494, AAEL014562, AAEL017096, AAEL017516 |
| GO:0043043 | peptide biosynthetic process | 4.02E-33 | AAEL000010, AAEL000823, AAEL000951, AAEL000987, AAEL001759, AAEL002372, AAEL002534, AAEL003582, AAEL003942, AAEL004151, AAEL004175, AAEL004503, AAEL005085, AAEL005097, AAEL005266, AAEL005451, AAEL005629, AAEL005817, AAEL005901, AAEL006511, AAEL006785, AAEL006860, AAEL007699, AAEL008083, AAEL008192, AAEL009151, AAEL009496, AAEL009747, AAEL009994, AAEL010168, AAEL011447, AAEL011471, AAEL011656, AAEL012733, AAEL012944, AAEL013221, AAEL013272, AAEL014494, AAEL014562, AAEL017096, AAEL017516 |
| GO:0034645 | cellular macromolecule biosynthetic process | 1.40E-16 | AAEL000010, AAEL000823, AAEL000951, AAEL000987, AAEL001324, AAEL001759, AAEL002372, AAEL002534, AAEL003582, AAEL003942, AAEL004151, AAEL004175, AAEL004503, AAEL005085, AAEL005097, AAEL005266, AAEL005451, AAEL005480, AAEL005629, AAEL005817, AAEL005901, AAEL006511, AAEL006785, AAEL006860, AAEL007022, AAEL007042, AAEL007699, AAEL008083, AAEL008141, AAEL008192, AAEL008953, AAEL009151, AAEL009496, AAEL009994, AAEL010168, AAEL011371, AAEL011447, AAEL011471, AAEL011656, AAEL012733, AAEL012944, AAEL013221, AAEL013272, AAEL014494, AAEL014562, AAEL017096, AAEL017516 |
| **GO:0006412** | **translation** | **2.40E-33** | **AAEL000010, AAEL000823, AAEL000951, AAEL000987, AAEL001759, AAEL002372, AAEL002534, AAEL003582, AAEL003942, AAEL004151, AAEL004175, AAEL004503, AAEL005085, AAEL005097, AAEL005266, AAEL005451, AAEL005629, AAEL005817, AAEL005901, AAEL006511, AAEL006785, AAEL006860, AAEL007699, AAEL008083, AAEL008192, AAEL009151, AAEL009496, AAEL009747, AAEL009994, AAEL010168, AAEL011447, AAEL011471, AAEL011656, AAEL012733, AAEL012944, AAEL013221, AAEL013272, AAEL014494, AAEL014562, AAEL017096, AAEL017516** |
| GO:0055114 | oxidation-reduction process | 3.72E-02 | AAEL018658, AAEL018687 |
| GO:0015980 | energy derivation by oxidation of organic compounds | 4.12E-03 | AAEL006721, AAEL018658, AAEL018662, AAEL018669, AAEL018685 |
| GO:0045333 | cellular respiration | 2.01E-03 | AAEL006721, AAEL018658, AAEL018662, AAEL018669, AAEL018685 |
| GO:0009060 | aerobic respiration | 2.42E-02 | AAEL006721, AAEL018662, AAEL018669 |
| **GO:0022900** | **electron transport chain** | **1.44E-03** | **AAEL018658, AAEL018664, AAEL018669, AAEL018685** |
| GO:0022904 | respiratory electron transport chain | 1.18E-02 | AAEL018658,AAEL018669,AAEL018685 |
| GO:0046034 | ATP metabolic process | 2.31E-02 | AAEL001668, AAEL018658, AAEL018668, AAEL018685 |
| GO:0006119 | oxidative phosphorylation | 2.05E-02 | AAEL018658 |
| GO:0042773 | ATP synthesis coupled electron transport | 2.05E-02 | AAEL018658 |
| GO:0042775 | mitochondrial ATP synthesis coupled electron transport | 1.48E-02 | AAEL018658 |
| GO:0006120 | mitochondrial electron transport, NADH to ubiquinone | 9.12E-03 | AAEL018658 |
