# Supplementary figures and images for: The Transcriptional Response of Aedes aegypti with Variable Extrinsic Incubation Periods for Dengue Virus
Source: Genome Biol Evol. 2018 Oct 18;10(12):3141–51. doi: 10.1093/gbe/evy230 (PMC6278894; doi:10.1093/gbe/evy230)

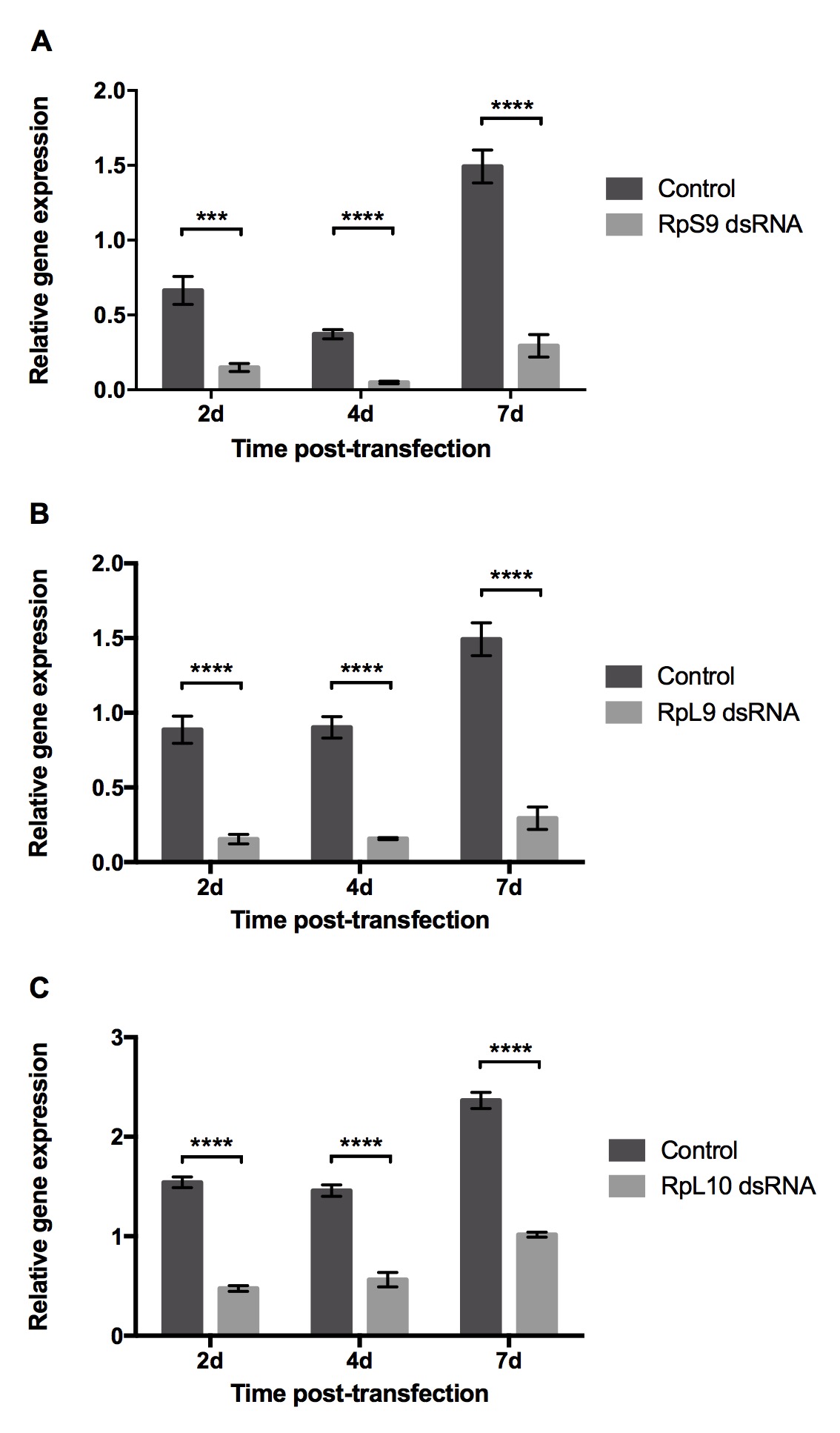

Supplement: Supplementary Data [file evy230_supp.zip › Supp Figure1.jpg]

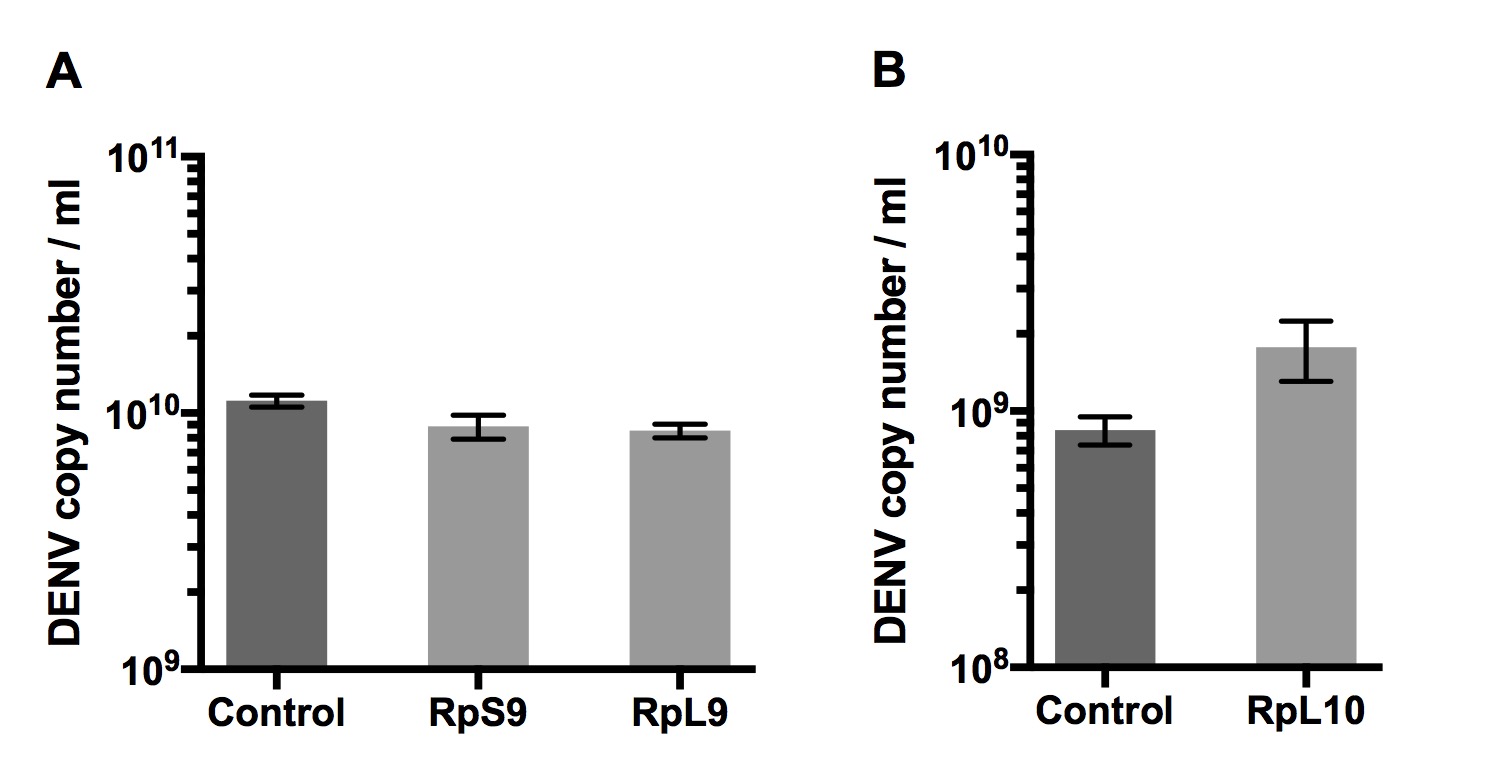

Supplement: Supplementary Data [file evy230_supp.zip › Supp Figure2.jpg]
